# Introducing a qualitative space

**DOI:** 10.1007/s40037-017-0331-7

**Published:** 2017-03-13

**Authors:** Lara Varpio, Elise Paradis, Chris Watling

**Affiliations:** 1 Department of Medicine, Uniformed Services University of the Health Sciences, Bethesda, MD USA; 2 Leslie Dan Faculty of Pharmacy, University of Toronto, Toronto, Canada; 3 Departments of Clinical Neurological Sciences, Oncology and Family Medicine at the Schulich School of Medicine and Dentistry, Western University, London, Canada

‘*Man cannot discover new oceans unless he has the courage to lose sight of the shore.*’ – André Gide

Health professions education is a relatively young field of inquiry; oceans of research questions and ideas await exploration by curious and creative scholars. Studying the role of emotions in clinical learning; tracing an individual’s professional identity development; investigating how competency and expertise are related but also importantly different: these are just some of the topics currently being explored in the health professions education literature. To chart these oceans, scholars rely on quantitative and qualitative methods and methodologies, crafting study designs from these traditions to best answer their research questions and to develop new knowledge.

And yet, since qualitative research is a more recent addition to the skill sets of many health professions education scholars, its methods and methodologies are sometimes perceived as difficult to use, challenging to review, and gruelling to get published. The qualitative methods and methodologies that have been frequently used in health professions education research – such as grounded theory, to give only one example – have found broad acceptance. Many introductory-level publications describe, for instance, the epistemological roots of qualitative research, how to do semi-structured interviews, and the development and growth of discourse analysis. However, there exists a broad range of qualitative approaches that have yet to be widely used or published in the health professions education literature. Reflecting on Andre Gide’s quote, we contend that qualitative research in health professions education has stayed close to the shore. We know that there are many highly skilled qualitative researchers in the field who are ready to sail further into the qualitative depths and, in so doing, make new discoveries in the oceans of health professions education’s ideas and questions.

Thus, it is with great excitement that we announce the launch of a new publication type here at *Perspectives in Medical Education *named *A Qualitative Space*. *A Qualitative Space* will highlight qualitative research approaches that push readers and scholars deeper into the waters of these methods and methodologies. With hopes of attracting intermediate- and advanced-level qualitative scholars, *A Qualitative Space *offers a place to, among other things: advance new ideas about qualitative methodologies, methods, and/or techniques; debate current and historical trends in qualitative research; craft and share nuanced reflections on how data collection methods should be revised or modified; reflect on the epistemological bases of qualitative research; or argue that some qualitative practices should end. These are papers that celebrate the many varieties of qualitative research, critique the qualitative methodologies and methods used unreflexively by scholars, and encourage creative experimentation with qualitative research approaches. In other words, *A Qualitative Space *will encourage qualitative scholars to lose sight of the shore and chart a new course.

*A Qualitative Space *will publish three types of content. First, when an empirical study using innovative qualitative methods or methodologies is accepted for publication in *Perspectives in Medical Education, *we (the editorial team leading *A Qualitative Space*) will review the manuscript and consider inviting a scholarly companion piece to illuminate the paper’s cutting-edge methodology, method, techniques, or design. Second, we will occasionally invite internationally recognized qualitative scholars to contribute an article for publication in the* A Qualitative Space* section. Finally, we encourage you, the health professions education scholars who are interested in qualitative research, to consider submitting methodology, methods, techniques and reflexively focused papers to this publication type. While this will not be a publication type for empirical research, it is a place to reflect upon that work and the qualitative approaches used therein. A full description of *A Qualitative Space *guidelines for authors is available in the journal’s Instructions for Authors.

In this edition of *Perspectives on Medical Education, *we are excited to publish the inaugural *A Qualitative Space* manuscript. In this paper, Dr. Carol Gilligan discusses the Listening Guide method – a voice-centred method of qualitative data analysis that she developed in the early 1980s and that has been widely adopted and celebrated by social scientists.

We hope that you are as excited about this new publication type as we are, and so will join us on this journey into qualitative research’s depths.

